# Melatonin levels in the Alzheimer’s disease continuum: a systematic review

**DOI:** 10.1186/s13195-021-00788-6

**Published:** 2021-02-23

**Authors:** Amber Nous, Sebastiaan Engelborghs, Ilse Smolders

**Affiliations:** 1 Research group Experimental Pharmacology (EFAR), Department of Pharmaceutical Chemistry, Drug Analysis and Drug Information (FASC), Center for Neurosciences (C4N), Vrije Universiteit Brussel, Laarbeeklaan 103, 1090 Brussels, Belgium; 2 Department of Neurology, UZ Brussel, Center for Neurosciences (C4N), Vrije Universiteit Brussel, Laarbeeklaan 101, 1090 Brussels, Belgium; 3 Reference Center for Biological Markers of Dementia (BIODEM), Institute Born-Bunge, University of Antwerp, Universiteitsplein 1, 2610 Antwerp, Belgium

**Keywords:** Alzheimer’s disease, Melatonin, Disruptions

## Abstract

**Background:**

The search for new Alzheimer’s disease (AD) cerebrospinal fluid (CSF) and blood biomarkers with potential pathophysiological and clinical relevance continues, as new biomarkers might lead to improved early and differential diagnosis, monitoring of disease progression and might even identify new druggable targets. Melatonin might be an interesting biomarker as an inverse correlation between CSF melatonin levels, and severity of the neuropathology as measured by Braak stages has been described. Melatonin can be measured in different body fluids, such as CSF, blood, saliva and urine.

**Objectives:**

The aim of this systematic review was to review all available studies regarding melatonin levels in different body fluids in the AD continuum and give an extensive overview of reported outcomes.

**Methods:**

We included papers comparing melatonin levels between healthy controls and human patients belonging to the AD continuum. A systematic search of PubMed and Web of Science led to inclusion of 20 full-length English papers following exclusion of duplicates.

**Results:**

This systematic literature search showed that disruptions in melatonin levels occur with age, but also in AD when compared to age-matched controls. Night-time melatonin levels were found to be lower in CSF and blood of AD patients as compared to controls. Literature was not conclusive regarding alterations in blood daytime melatonin levels or regarding saliva melatonin in AD patients. Decreased total and night-time melatonin production has been described in urine of AD patients.

**Conclusion:**

Our systematic review shows evidence for disruptions in (night-time) melatonin levels in AD as compared to age-matched controls. Although more studies are needed to understand the contribution of disruption of the melatonergic system to the pathophysiology of AD, the potential anti-AD effects that have been attributed to melatonin, renders research on this topic relevant for the discovery of potential future treatment effects of melatonin for AD. The use of melatonin as potential blood biomarker for disease progression should also be further investigated.

## Background

Worldwide, around 50 million people suffer from dementia, with nearly 10 million new cases every year. Alzheimer’s disease (AD), a currently incurable condition, is the most prevalent cause of dementia, accounting for 60–70% of cases [1, 2]. In AD, the pathophysiological changes begin many years prior to the first clinical manifestations of the disease. AD is a continuum, including asymptomatic individuals to patients with severe dementia [3]. Different biomarkers, amongst which cerebrospinal fluid (CSF) amyloid-β (Aβ42), total tau (T-tau) and phosphorylated tau (P-tau), are used for early and differential AD diagnosis [4, 5]. As blood sampling is less invasive than a lumbar puncture, blood biomarkers for AD are under development [5]. The search for new CSF and blood biomarkers with potential pathophysiological and clinical relevance continues, as new biomarkers might lead to improved early and differential diagnosis, monitoring of disease progression and might even identify new druggable targets.

Melatonin is a neurohormone, mainly produced by the pineal gland under influence of the hypothalamic suprachiasmatic nucleus (SCN), regulating circadian rhythms such as the sleep-wake rhythm, neuroendocrine rhythms and body temperature cycles [6]. Levels of melatonin in humans are low during the day and peak during the night in healthy adults [7]. Melatonin concentrations have mainly been studied in blood. However, melatonin can also be measured in other body fluids such as CSF, urine, and saliva. Melatonin concentrations tend to be high in the third ventricle in which it is directly secreted from the pineal gland via the pineal recess [8]. Being an amphiphilic neurohormone, melatonin can access neurons nearby the third and lateral ventricles [9]. Away from the third ventricle, there is a concentration gradient with levels dropping in spinal CSF to about the same amount as found in blood [10]. The use of urine and saliva for measuring melatonin has the benefit of being less invasive and being able to be done at home.

Melatonin levels have been studied in these different body fluids in patients with AD in different studies. Studying melatonin levels in neurological diseases might be interesting, since melatonin seems to have neuroprotective effects [6]. Melatonin has anti-inflammatory, anti-oxidant, anti-fibrillogenic, anti-hyperphosphorylating and anti-amyloidogenic pathway properties [11], and CSF melatonin levels have been shown to negatively correlate with Braak stages in AD [12], making it particularly interesting to study melatonin levels in AD, not only to assess whether it could be useful as treatment strategy in AD but also to pave the way to more studies with blood melatonin as potential biomarker to track disease progression, if disrupted melatonin production in AD were to be proved.

The aim of this systematic review was to formulate an answer to the following question: ‘How are melatonin levels altered in subjects belonging to the AD continuum as compared to healthy controls in different body fluids?’

## Methods

In order to find relevant publications about melatonin levels in the AD continuum, a systematic literature search was conducted. We selected studies that reported melatonin levels in subjects belonging to the AD continuum as compared to controls. Since most initially screened studies about melatonin levels were old, older standard clinical diagnostic criteria for AD were accepted for inclusion in this systematic review. We provided broad coverage of patient eligibility, including patients eligible based on National Institute on Aging and Alzheimer Association (NIA-AA) criteria [13, 14], National Institute of Neurological and Communicative Disorders and Stroke and the Alzheimer’s Disease and Related Disorders Association (NINCDS-ADRDA) criteria [15], Diagnostic and Statistical Manual for Mental Disorders (DSM) III and IV criteria [16, 17] or Petersen criteria for MCI [18].

This search was done between October 2019 and October 2020. The following electronic databases were used: PubMed and Web of Science. These databases provide broad coverage of medical and biological journals worldwide. Combinations of search terms ‘melatonin’ and ‘Alzheimer’ were used in order to have a broad coverage of the topic. We limited the search to articles available in English. There were no limitations regarding publication date of the article. Additionally, the bibliographies of published studies were also used to search for potentially relevant articles regarding melatonin levels in AD.

**Fig. 1:**
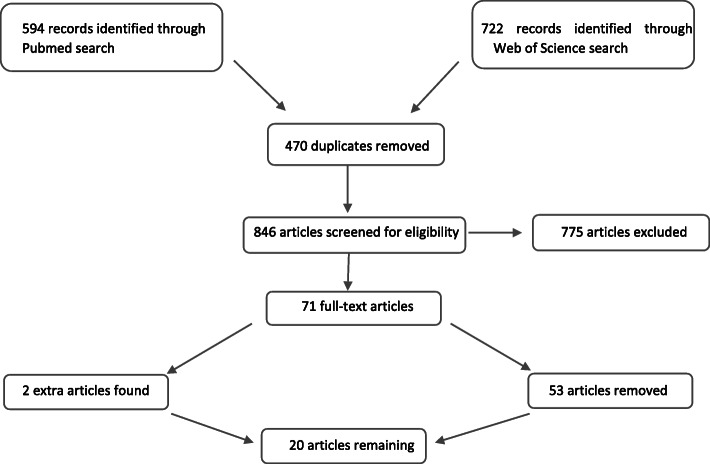
Study flow diagram

In PubMed, the search using ‘melatonin’ and ‘Alzheimer’ lead to 594 hits, in Web Of Science to 722 hits. Four hundred seventy duplicates were removed by use of Mendeley, leaving us with 846 articles. Articles were screened based on title and abstract, with removal of articles regarding melatonin in animals (as the majority of laboratory mouse strains (e.g. C57BL/6 J, CF-1, 129/Sv, BA/2, BALB/c) do not produce melatonin, except for the CBA/Ms and C3H/He strains, due to genetic backgrounds with absence of N-acetyltransferase (NAT) and/or hydroxyindole-O-methyltransferase (HIOMT) enzyme activity that make the mice melatonin deficient [19, 20]), articles without description of melatonin levels in body fluids (e.g. about melatonin as potential therapeutic target), articles in other languages than English, articles that mention melatonin levels in other diseases than AD or articles where melatonin was not compared between patients with AD and healthy controls. Editorial letters or probable wrong references after checking with our internal library were also excluded, leaving us with 71 full-text articles. After reading these articles, 53 articles were removed because they too did not report melatonin levels in body fluids in AD as compared to controls. Based on the bibliographies of read articles, 2 extra articles were found and used, leaving us with 20 articles in total. The study flow diagram can be found in Fig. 1. The Newcastle-Ottawa Scale (NOS) was used for assessing the quality of the articles included [21]. This scale can lead to a maximum score of 9 for each individual study, with studies with increasing NOS being considered as having increased quality.

## Results

### CSF

Melatonin is secreted into the third ventricle directly via the pineal recess. There is a concentration gradient in CSF melatonin away from the third ventricle [10]. Wu et al. measured melatonin levels in pineal glands but also in post-mortem ventricular CSF, obtained at autopsy, in a subgroup of a clinically and pathologically confirmed AD cohort. They found a highly positive correlation between CSF melatonin levels and pineal melatonin levels, suggesting a good reflection of pineal melatonin by CSF melatonin (*r* = 0.83, *P* < 0.0001) [22]. An overview of findings regarding CSF melatonin can be found in Table 1.

**Table 1:**
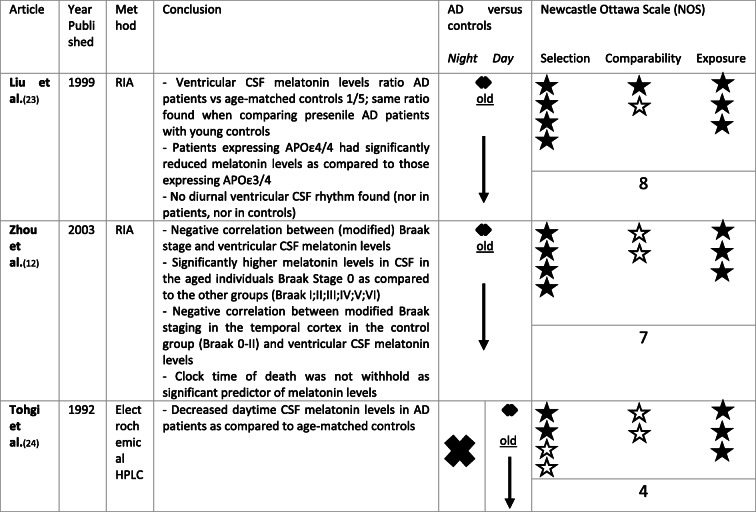
Overview of CSF melatonin levels in AD as compared to controls. ↓ means a significant reduction in CSF melatonin in AD when compared to controls. ✖ means no data comparing CSF melatonin levels between AD and controls were available. A black star ★ was attributed per NOS criterium per category (selection, comparability, exposure) met. Stars were kept white ☆ if the NOS criterium in the corresponding category was not met

#### Day- and night-time melatonin levels

Post-mortem ventricular CSF melatonin levels were determined in clinically and pathologically defined AD patients and compared to age-matched controls by Liu et al. Within the control group, melatonin levels were half in those aged > 80 years as compared to those aged < 80 years (*p* < 0.01). Furthermore, melatonin levels in AD patients were 1/5 of the levels measured in the age-matched control group (*p* = 0.0001). The early-onset AD patients (< 65 years old) also presented with melatonin levels 5 times lower than the young controls, with similar melatonin levels to the senile AD group, suggesting a role of the disease and not only of age in the melatonin disruptions. AD patients carrying two APOε4 alleles furthermore had significantly decreased melatonin levels as compared to those carrying the APOε3/4 genotype. They could not find a diurnal rhythm (day- vs night-time difference) in CSF melatonin levels in the control or AD group [23]. Zhou et al. measured melatonin in post-mortem ventricular CSF of patients with definite AD Braak stage V–VI (mean age: 77 +/− 2 years), Braak stage III–IV (mean age: 87 +/− 2 years) and controls without neurological or psychiatric disease but also including subjects in Braak stage I and II (mean age: 76 +/− 2 years). They found that post-mortem CSF melatonin levels negatively correlated with classical Braak stage (0–VI), with significantly higher melatonin levels in CSF in the aged individuals Braak stage 0 as compared to the other groups (*p* < 0.03). Melatonin levels also negatively correlated with Modified Braak stage in the entire cortex (in which each cortical area—frontal, temporal, parietal, occipital—was evaluated separately for neuropathological changes such as neuritic plaques, NFTs and neuripil disruptions). Furthermore, they found a significant negative correlation between CSF melatonin and (modified) Braak staging in the temporal cortex in the control group (Braak stage 0–II): CSF melatonin levels were 7 times higher in those without neuritic plaques than those with and 3 times higher in those without NFTs than those with. These findings suggest that decreases in melatonin might be present in early stages of the disease, even before occurrence of clinical symptoms. The multiple linear regression model did not withhold clock time of death as significant predictor of melatonin levels [12]. These findings support CSF melatonin disruptions in AD patients, and suggest a potential role for the AD genetic risk factors in disrupted melatonin production.

#### Daytime melatonin levels

Tohgi et al. found significantly decreased melatonin levels in lumbar CSF, measured between 09 and 10 AM, in AD patients as compared to age-matched controls. The diagnosis of AD was made according to DSM III-R, NINCDS-ADRDA criteria, and furthermore based on CT and MRI findings. However, reported melatonin concentrations are high in comparison with other studies (range of ng/ml instead of pg/ml) possibly due to inaccurate measurement [24].

### Blood

Melatonin levels in AD are mostly studied in blood, probably due to its less invasive nature as compared to CSF which requires lumbar puncture. In healthy adults, serum and CSF melatonin was correlated [25]. This correlation has, to our knowledge, not yet been shown in AD patients. An overview of findings regarding blood melatonin can be found in Table 2.

**Table 2:**
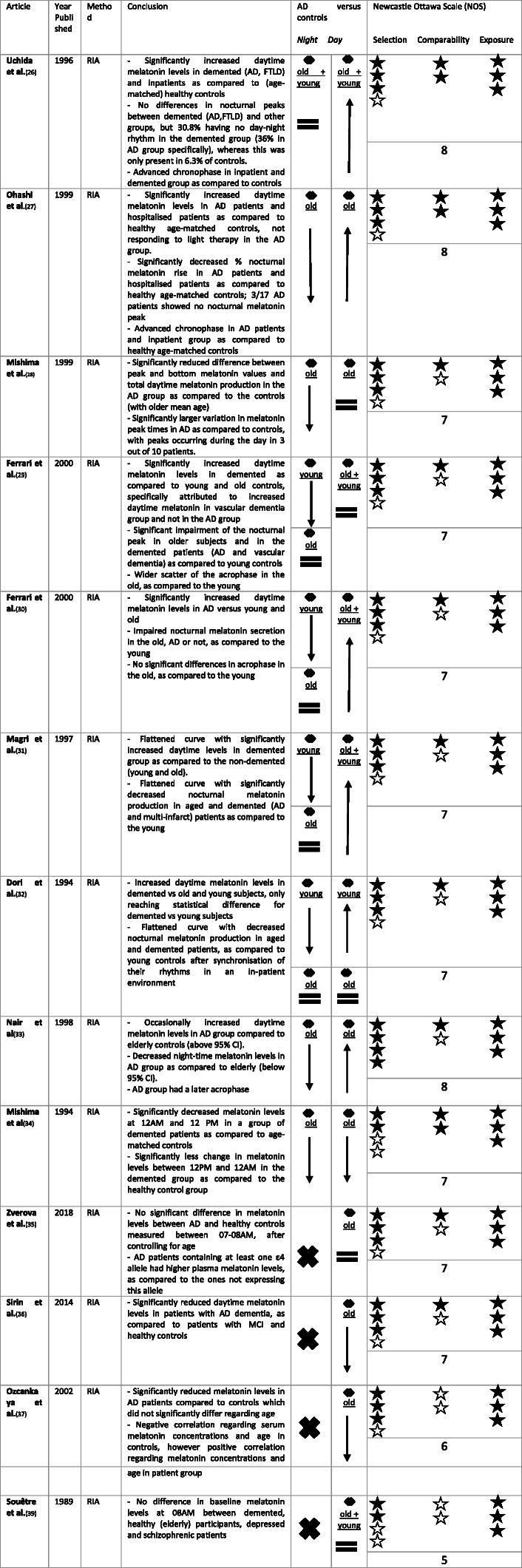
Overview of blood melatonin levels in AD as compared to controls. ↓ means a significant reduction in blood melatonin in AD when compared to controls. ↑ means a significant increase in blood melatonin in AD when compared to controls. = means no significant difference in pineal melatonin in AD when compared to controls. ✖ means no data comparing blood melatonin levels between AD and controls were available. A black star ★ was attributed per NOS criterium per category (selection, comparability, exposure) met. Stars were kept white ☆ if the NOS criterium in the corresponding category was not met

#### Daytime melatonin levels

Uchida et al. measured serum melatonin levels every 3 h in a group of 13 hospitalised, demented patients (with 11 of them having AD and 2 of them FTLD, diagnosis based on DSM-III-R and NINCDS-ADRDA criteria, as well as CT findings), a group of hospitalised patients for other than dementia causes, a group of age-matched healthy controls and a group of young volunteers. The former two going to bed at 19:00 and waking up at 06:30 with exposition to low light intensities during the day (max. 100 lx), the latter two going to bed at 23:00–24:00 and waking up at 07:00–08:00 with exposition to high light intensities during the day (5000–20,000 lx). Increased daytime melatonin levels were found in the demented and inpatient group as compared to healthy controls (young and age-matched) [26]. Ohashi et al. also found significantly increased daytime melatonin levels in AD patients diagnosed according to NINCDS-ADRDA and DSM-IV criteria and in an age-matched hospitalised group as compared to the age-matched control group. Furthermore, the daytime levels in AD patients did not decrease after light exposure treatment, whereas this was effective for the reference inpatient group [27]. Mishima et al. compared serum melatonin levels, measured every 2 h, between patients with AD according to NINCDS-ARDRA criteria who had sleep and behavioural disorders and non-demented controls without clinical sleep disorders, both staying at different wards in the same facility. They reported decreased total daily (over 24 h) melatonin production (area under the curve [AUC]) in the AD group as compared to the controls, as well as reduced difference between peak and bottom melatonin values; however, no difference in daytime melatonin levels was reported [28]. Ferrari et al. found that a group of patients with dementia (AD according to NINCDS-ADRDA and multi-infarct dementia according to Hachinski ischemic score) had increased daytime levels as compared to the young and old controls but this was especially the case for patients with multi-infarct dementia and not for the AD group [29]. Another study by Ferrari et al., however, showed increased daytime melatonin levels in the AD group (AD according to DSM-III-R and NINCDS-ADRDA criteria with Hachinski ischemic score of less than 4, diagnosis furthermore supported by brain imaging) as compared to elderly and young controls [30]. Magri et al. found a flattened melatonin curve in a demented group (AD according to NINCDS-ADRA or multi-infarct dementia according to Hachinski ischemic score) as compared to the non-demented elderly with higher daytime levels in the demented group [31]. Dori et al. also found daytime melatonin levels of the old demented (AD and multi-infarct dementia) patients to be significantly higher at 12:00, 16:00 and 20:00 as compared to the young controls. Furthermore, at 16:00 and 20:00 these levels tended to be higher in old demented than in old intact patients without reaching statistical differences [32]. Nair et al. found daytime melatonin levels of AD patients according to NINCDS-ADRDA to be occasionally above the 95% upper confidence interval (CI) of normal elderly [33]. In a group of patients with multi-infarct dementia or AD with insomnia or sleep-wake schedule disorder, the effect of bright light treatment during 2 h in the morning on melatonin levels was evaluated. Before treatment, and in contrast to previously mentioned findings, the melatonin levels were significantly lower at 12 AM (and 12 PM) than their age-matched control group. They did not find a significant change in melatonin levels during or after the light therapy [34]. More recently, Zverova et al. could not find a statistical difference in plasma melatonin levels between AD patients (meeting NINCDS-ADRDA criteria) and controls, taken between 07 and 08 AM, after controlling for age. They found that plasma melatonin levels were increased in the group of AD patients containing at least one ε4 allele, as compared to the ones not expressing this allele. This is in contrast with the negative influence of APOε4/ε4 genotype found by Liu et al. [23, 35] Sirin et al. compared serum melatonin levels, taken between 08 and 09 AM, between patients with AD dementia as defined by DSM IV criteria, MCI as defined by Petersen criteria and healthy controls. They found significantly lower levels in the patients with AD dementia as compared to the MCI and healthy control group, further existing when correcting for age [36]. In line with these findings, Ozcankaya et al. performed a study measuring different antioxidants between 07 and 08 AM in patients with AD according to DSM-IV criteria and in healthy controls. Patients and controls did not significantly differ regarding age or sex. The AD group had significantly reduced melatonin levels as compared to the control group (*p* < 0.001) [37]. Souêtre et al. suggest functional impairment of the pineal gland in dementia, as suggested before, but not in depression and in healthy (elderly) subjects. Their previous work showed that 5-methoxypsoralen was able to induce a dramatic surge of melatonin after either morning or evening administration in humans [38]. In this study, they found an impaired melatonin response 3 h after administration of 5-methoxypsoralen in patients with dementia, selected according to DSM III criteria. All other groups, including healthy young, healthy middle-aged, healthy elderly, depressed patients and schizophrenic patients showed a significant surge in plasma melatonin levels 3 h after administration. The demented patients did not significantly differ regarding age with the healthy elderly subjects. The baseline melatonin levels of demented patients at 08 AM, before 5-methoxypsoralen administration, did not differ from other groups [39]. We could conclude that regarding daytime blood melatonin levels literature is inconclusive, with increased as well as decreased or similar levels reported in literature compared to control subjects.

#### Night-time melatonin levels

Uchida et al.*,* who measured serum melatonin levels every 3 h as mentioned in the previous paragraph, found no significant difference in peak levels recorded at night between the different groups; however, 4 out of 13 demented patients did not show a nocturnal increase, whereas this was only the case for 2 out of 32 controls. In the AD group specifically, 36% did not show a diurnal melatonin rhythm. Furthermore, they found an advanced chronophase with early night occurrence of the peak melatonin levels in the inpatient and demented group [26]. Ohashi et al. also suggest an advanced chronophase in patients with AD and in a hospitalised age-matched reference group compared to an age-matched control group: when measuring melatonin levels every 3 h, there was a significant increase in melatonin levels at 21:00, 24:00 and 3:00 in the AD and hospitalised group as compared to the other time points whereas this increase was seen at 24:00, 3:00 and 6:00 in the control group. The percentage of nocturnal rise was significantly decreased in the AD and hospitalised group as compared to the control group. Three out of 17 AD patients did not show a nocturnal increase [27]. The study by Mishima et al. showed that the AD patients had decreased peak night-time melatonin levels as compared to the non-demented controls with older mean age, with significantly decreased total daily melatonin production. They showed a large variation in peak times, with 3 out of 10 AD patients having a daytime melatonin peak [28]. Ferrari et al. also showed significant impairment of the nocturnal peak in older subjects and in the demented patients (AD and vascular dementia) as compared to young controls. In line with previous findings, there was a wider scatter of the acrophase in the old and demented [29]. The other study by Ferrari et al. showed similar findings with an impairment in the nocturnal melatonin peak in elderly and demented (AD) as compared to the young [30]. Magri et al. also found significantly decreased nocturnal melatonin secretion in a group of elderly patients, demented or not, as compared to the young subjects [31]. Dori et al. found significantly reduced nocturnal melatonin production in elderly patients, demented and cognitively intact, as compared to young controls [32]. Nair et al. found night-time melatonin levels of patients with AD to be below the 95% lower CI of normal elderly with lower peak night-time secretion. Acrophase occurred later in the AD than non-AD group [33]. As mentioned before, demented patients with multi-infarct dementia or AD had significantly decreased melatonin levels at 12 PM as compared to an age-matched control group [34]. Night-time melatonin levels thus seem to be consistently lower in elderly, and in some studies, even more specifically in AD patients compared to age-matched controls.

### Saliva

In recent years, there is growing interest in saliva melatonin levels, since saliva collection is not invasive and can be done at home. In blood, melatonin is bound to albumin and alpha-1-acid glycoprotein. The melatonin in saliva represents the unbound blood melatonin levels. Melatonin levels in saliva are estimated to be approximately 30% of the total blood melatonin concentrations. This means that daytime saliva levels are low, even less than 2 pg/ml [40]. An overview of findings regarding saliva melatonin can be found in Table 3.

**Table 3:**
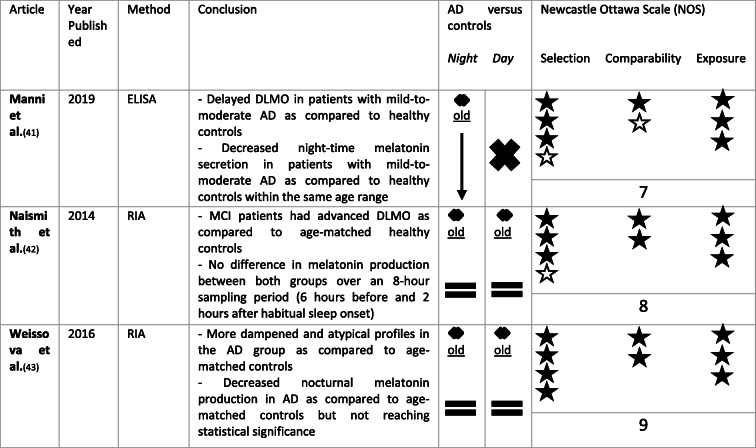
Overview of saliva melatonin levels in AD as compared to controls. ↓ means a significant reduction in saliva melatonin in AD when compared to controls. = means no significant difference in saliva melatonin in AD when compared to controls. ✖ means no data comparing saliva melatonin levels between AD and controls were available. A black star ★ was attributed per NOS criterium per category (selection, comparability, exposure) met. Stars were kept white ☆ if the NOS criterium in the corresponding category was not met

Manni et al. selected AD patients according to more recent NIA-AA criteria with dementia from mild to moderate severity (Mini-Mental State Examination [MMSE] 14–24) and selected healthy controls within the same age range. All participants collected five saliva samples in a dark (< 10 lx) environment. They calculated the dim-light melatonin onset (DLMO), which is the rise in melatonin in the evening in the absence of light. A delayed DLMO in patients with mild-to-moderate AD as compared to healthy controls was found. Furthermore, melatonin secretion following DLMO was significantly decreased in the AD patients as compared to the healthy controls. No differences regarding DLMO and melatonin levels were found when comparing patients with MMSE > 18 and MMSE ≤ 18 [41]. In contrast, Naismith et al. found that patients with MCI, as defined by Petersen criteria, had an advanced DLMO as compared to age-matched controls but showed no difference in the total amount of melatonin secreted over an 8-h sampling period (6 h before and 2 h after habitual sleep onset) [42]. Weissova et al. found a trend towards lower melatonin production during the night in the AD group as compared to age-matched controls [43].

### Urine

Approximately 85% of circulating melatonin is inactivated by the liver by CYP1A1, CYP1A2 and possibly CYP2C19 and is converted to 6-hydroxy-melatonin. After conjugation with sulphate, it is excreted in urine in a water-soluble form as 6-hydroxy-melatonin sulphate (aMT6s) [44]. An overview of findings regarding urine melatonin can be found in Table 4.

**Table 4:**
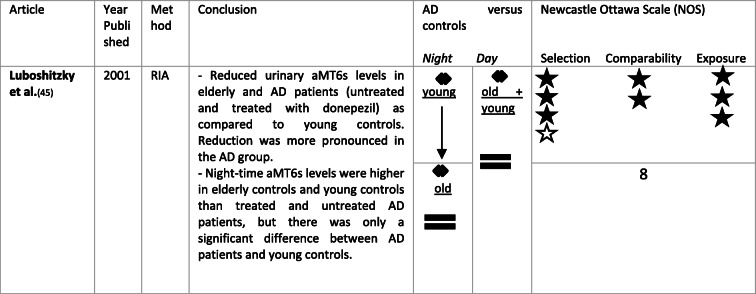
Overview of urine melatonin levels in AD as compared to controls. ↓ means a significant reduction in urine melatonin in AD when compared to controls. = means no significant difference in urine melatonin in AD when compared to controls. A black star ★ was attributed per NOS criterium per category (selection, comparability, exposure) met. Stars were kept white ☆ if the NOS criterium in the corresponding category was not met

Luboshitzky et al. measured aMT6s levels in untreated male AD patients living at home, AD patients treated with donepezil, and in age-matched elderly males and in young males. They found total aMT6s levels to be significantly reduced in elderly controls, in patients with untreated and treated AD as compared to young controls. A day-night difference in aMT6s secretion was seen in all young controls, in 50% of elderly controls, in only 20% of patients with untreated AD, and in 67% of those with donepezil treatment. Night-time aMT6s levels were lower in treated and untreated AD patients as compared to elderly controls and young controls, only reaching statistical difference in comparison with young controls. Daytime aMT6s levels did not significantly differ between these groups. These data are in line with previously mentioned findings in blood and CSF, with reductions in melatonin with age and even more pronounced reductions in AD. An interesting fact is that the day-night difference in melatonin production was more pronounced in AD patients treated with donepezil than those patients without treatment and than in cognitively normal elderly [45].

## Discussion

This systematic review suggests disruptions in melatonin production in the AD continuum, which is not solely explained by increasing age in this specific population [12, 23, 24, 27, 28, 33, 34, 41]. In CSF, all studies pointed towards decreased melatonin levels in AD patients when compared to aged controls [12, 23, 24]. No clear CSF day-night melatonin rhythm has been found in patients nor controls [23] and clock time of death was not withheld as significant predictor for CSF melatonin levels in a group of participants belonging to AD Braak stage 0–Braak stage VI [12]. The largest part of the studies regarding melatonin levels in AD has been performed using blood, probably because it is less invasive when compared to lumbar puncture. The studies regarding melatonin levels in blood also confirm altered melatonin production in AD with advanced chronophase or wider scatter of the acrophase, decreased nocturnal melatonin levels and absence of nocturnal melatonin peaks in AD patients, especially as compared to the young and in some studies as compared to age-matched [26–34]. When looking at Table 2, we could conclude that nocturnal melatonin blood levels significantly decrease with age [29–31], but might become even more impaired in AD patients [27, 28, 33, 34]. Regarding daytime blood melatonin levels, literature is contradictive with increased as well as decreased or unaltered levels reported in AD as compared to healthy controls [26–37, 39]. Saliva and urine melatonin levels are also shown to be disrupted in AD. Nocturnal saliva melatonin as well as nocturnal and total urinary aMT6s levels tended to decrease [41, 43, 45], whilst changes in chronophase were reported [41, 42] as compared to controls.

The studies by Uchida et al. [26] and Ohashi et al. [27] emphasise on environmental aspects to take into account when interpreting melatonin levels, e.g. daytime melatonin levels were increased in the demented group but also in a group of non-demented hospitalised patients, probably due to low light exposure as inpatient [26, 27]. Next to light exposure, other aspects to take into account when interpreting or performing studies with melatonin levels are the influence of medication (e.g. β-blockers) [46], position (standing vs sitting) [47], and presence of other pathologies [48].

Whereas the correlation between pineal gland melatonin and ventricular CSF melatonin has been confirmed in a cohort of AD patients [22], the correlation between blood melatonin and CSF melatonin has only been shown in a group of healthy controls [25] but should be further investigated in the AD continuum. A significant correlation between saliva and serum melatonin levels has also been shown in a group of healthy volunteers [49], as well as a significant correlation in time of onset of melatonin production and acrophase as measured by saliva melatonin versus plasma melatonin [50]. Furthermore, overnight melatonin secretion over a 16-h observation period, as measured by AUC in plasma correlated significantly with the amounts of urinary aMT6s and urinary melatonin excreted over that period [51]. To our knowledge, these correlations have not yet been found in the AD continuum and merit further investigation. An overview of validation studies can be found in Table 5.

**Table 5:** Validation for assessment of melatonin levels in one body fluid versus another. ‘V’ means validation exist, ‘?’ means that, to our knowledge, no validation exists

	AD continuum	Healthy volunteers
CSF versus pineal gland	**V**	**?**
Blood versus CSF	**?**	**V**
Saliva versus blood	**?**	**V**
Urine versus blood	**?**	**V**

Some studies investigated melatonin levels in post-mortem pineal glands of AD patients [22, 52]. Wu et al. found lower nocturnal melatonin levels within post-mortem pineal glands of AD patients but also in cognitively intact persons with minor AD neuropathological changes in the brain (Braak stage I–II) as compared to controls. The significant reduction in nocturnal pineal melatonin led to an overall loss of day-night rhythm in the study group with AD neuropathological changes [22]. This could explain the absence of day-night rhythm in CSF of AD patients [12, 23]. Skene et al. also reported significantly reduced nocturnal post-mortem pineal melatonin with a loss of day-night rhythm when comparing AD patients to controls, but disappearing when comparing AD patients to age-matched controls, thus attributing the loss to increasing age rather than disease [52]. The absence of day-night rhythm in CSF of controls could therefore be explained by old age of the control group [12, 23].

Underlying pathophysiological mechanisms of the disease might contribute to disrupted melatonin production. The hypothalamic SCN might get involved in the disease process with deposition of pretangles and tangles in the nucleus [53]. Furthermore, plaque pathology has been described in the hypothalamus (e.g. phase 3 of Thal stages), but not necessarily in the SCN [54–56]. A significant loss in SCN neuronal cell numbers (total, arginine vasopressin- [AVP-], vasoactive intestinal peptide- [VIP-] and neurotensin- [NT-] expressing) has been described [53, 57–60]. Involvement of the SCN in the disease process might lead to deficits in melatonin production. Furthermore, significantly reduced functional pineal volume (without calcifications and cysts), with higher degrees of calcifications in the pineal gland have been described in AD as compared to controls [61–63]. Another possible explanation for disruptions in melatonin production in AD might be deficiencies in melatonin’s precursor serotonin (5-HT) or decreases in norepinephrine, which is responsible for activating the pineal gland via *β-*receptors, due to increased MAO-A activity in AD. Wu et al. found increased MAO-A mRNA levels in the pineal gland of cognitively intact cases with minor AD neuropathologic changes (Braak stage I-II) and AD patients in Braak stage VI, as compared to controls in Braak stage 0. 5-Hydroxyindoleacetic acid (5-HIAA) levels, the oxidative product of 5-HT by MAO-A was also increased in the pineal gland of these groups as compared to Braak stage 0 [22].

Altered melatonin production might explain the circadian rhythm disturbances seen in AD. Sundowning syndrome and nightly restlessness are known causes for institutionalisation in AD patients [64]. Sleep disturbances, such as nightly restlessness and sundowning, according to the glymphatic hypothesis [65, 66], might lead to disease progression by decreased functioning of the glymphatic system responsible for drainage of solutes like amyloid beta (Aβ), or by a relative increase in neuronal activity with increased production of Aβ as a consequence [67]. Furthermore, reductions in melatonin levels might lead to disease progression by loss of its potential anti-AD effects [11]. Melatonin has anti-inflammatory and anti-oxidant effects, as well as anti-fibrillogenic, anti-hyperphosphorylating and anti-amyloidogenic pathway properties [11]. There is evidence from preclinical studies that melatonin has aforementioned effects in AD [68–72], amongst which a study in an Aβ vaccinated mouse model. Aβ vaccination is under investigation as treatment approach in AD, but inflammatory responses against Aβ fragments seem to be a limiting factor. Non-melatonin-treated Aβ vaccinated mice showed elevated levels of lipid peroxides (LPO), an oxidative stress marker, and decreased antioxidant enzyme levels, whereas this was normalised in the melatonin-treated Aβ vaccinated mice [68]. In another study, fibrillar Aβ (fAβ) was administered to the hippocampal CA1 region of rats, leading to increased oxidative stress markers, such as nitrites and LPO in brain homogenates. However, in melatonin-treated fAβ rats, nitrite and LPO levels were significantly reduced. Its anti-inflammatory effects were also confirmed by significantly reducing the IL-1β, IL-6 and TNF-levels as compared to the non-melatonin-treated group [69]. Pappolla et al. showed in an in vitro study that melatonin interacted with Aβ1–40 and Aβ1–42 and reduced amyloid fibril formation [70]. Glycogen synthase kinase 3 (GSK3) overactivity in AD induces hyperphosphorylation of tau [73] but seems to be targeted by melatonin therapy [71]. An in vitro study showed that 24 h melatonin treatment was able to significantly reduce endogenous BACE1 and presenilin 1 (subunit of y-secretase) at both protein and mRNA levels in human SH-SY5Y cells [74]. Furthermore, melatonin upregulates the α-secretases ADAM10 and ADAM17 catalytic activities and endogenous protein levels and induces their promoter transactivation, which leads to nonamyloidogenic processing of APP [72]. Melatonin also exhibits antidepressant properties, addressing another important comorbidity of AD with negative influence on cognitive functions [48].

We hypothesise that especially disruptions in night-time melatonin levels are part of the disease and might lead to disease progression. Night-time melatonin levels generally tend to be much higher than daytime melatonin levels making decreased night-time melatonin production more susceptible for having influence on disease progression. Ferrari et al. found an inverse correlation between the severity of dementia and the melatonin nocturnal peak [29]. However, Manni et al. did not find significant differences in night-time melatonin levels between patients with MMSE > 18 and ≤ 18 [41]. Sirin et al. found an inverse correlation between daytime melatonin levels and MMSE scores [36], suggesting a role for daytime melatonin levels as well, but, as said before, there is no clear consensus about daytime melatonin levels in AD. These links between melatonin levels and scoring on cognitive tests merit further investigation to elucidate on melatonin’s effects on cognition. Retrospective studies and a meta-analysis showed that melatonin administration in patients with MCI according to Petersen criteria, but not in patients with AD had positive effects on cognition [75–77]. In AD dementia, it only exerted positive effects on sleep time at night, which was also seen in the MCI cohort. The question remains whether the positive influence of melatonin on cognition found in these MCI patients cannot solely be explained by patient’s better sleep quality when being under melatonin treatment as shown in these studies. Further studies with melatonin administration in patients with MCI, or even in patients with preclinical AD, without pre-existing sleep disturbances, would be useful.

Not only might low night-time melatonin levels lead to disease progression as discussed above, Zhou et al. furthermore found that post-mortem CSF melatonin levels negatively correlated with Braak stages (0–VI) [12]. Since the development of blood biomarkers to track AD progression has been proven to be difficult [5] and night-time blood melatonin levels also seem to be disrupted in AD [27, 28, 33, 34], it might be interesting to study whether blood melatonin levels have the potential to be used as biomarker for disease progression in AD.

## Conclusions

Based on our systematic search, we conclude that melatonin levels in CSF, blood, saliva and urine are altered in elderly people and might even become more impaired in AD patients as compared to controls, possibly due to significantly decreased SCN cell number and functional pineal gland volume. The typical diurnal melatonin rhythm disappeared in CSF of elderly subjects and AD patients. Findings were conclusive regarding night-time melatonin concentrations where the majority of studies pointed to decreased melatonin levels in AD versus healthy persons. However, regarding daytime blood melatonin levels, findings were inconsistent. With regard to melatonin disruption’s negative influence on disease progression, we hypothesise that especially the decreased night-time melatonin levels are important. These levels are higher in concentration than daytime melatonin levels, potentially exerting larger influences on the brain. Regarding saliva and urine melatonin levels, a limited number of studies have been published, but they point towards decreases in (night-time) melatonin levels. Further studies are needed to conclude on changes in blood daytime, saliva and urine melatonin levels in AD as compared to controls, as well as on the validation of blood, saliva and urine melatonin as compared to CSF in patients with AD. Night-time melatonin levels seem to be significantly and consistently low in AD patients which might pave the way to further research with melatonin as possible new (early) treatment strategy, not only to prevent sundowning and sleep disturbances, but potentially also to reduce cognitive decline in this currently incurable condition. The use of melatonin as potential blood biomarker for disease progression should be further investigated.

## Data Availability

All data generated during this study/systematic review are included in this published article since this data only includes information acquired from published studies (see references).

## Abbreviations

AD: Alzheimer’s disease
SCN: Suprachiasmatic nucleus
5-HT: Serotonin
CSF: Cerebrospinal fluid
MCI: Mild cognitive impairment
MAO: Monoamine oxidase
Aβ: Amyloid beta
LPO: Lipid peroxides
fAβ: Fibrillar amyloid beta
GSK3: Glycogen synthase kinase 3
NIA-AA: National Institute on Aging and Alzheimer Association
NINCDS-ADRDA: National Institute of Neurological and Communicative Disorders and Stroke and the Alzheimer’s Disease and Related Disorders Association
DSM: Diagnostic and Statistical Manual for Mental Disorders
NAT: N-acetyltransferase
HIOMT: Hydroxyindole-O-methyltransferase
NOS: Newcastle Ottawa Scale
VIP: Vasoactive intestinal peptide
AVP: Arginine vasopressin
NT: Neurotensin
NFT: Neurofibrillary tangles
FTLD: Frontotemporal lobar degeneration
CT: Computed tomography
MRI: Magnetic resonance imaging
AUC: Area under the curve
CI: Confidence interval
MMSE: Mini-Mental State Examination
DLMO: Dim Light Melatonin Onset
aMT6s: 6-hydroxy-melatonin sulphate
